# Gold nanomaterials in the management of lung cancer

**DOI:** 10.1042/ETLS20200332

**Published:** 2020-12-03

**Authors:** Ainoa Guinart, Hannah L. Perry, James D. E. T. Wilton-Ely, Teresa D. Tetley

**Affiliations:** 1 National Heart and Lung Institute, Imperial College London, London, U.K.; 2 Department of Chemistry, Imperial College London, London, U.K.

**Keywords:** diagnosis, gold nanoparticles, imaging, lung cancer, nanomedicine, therapy

## Abstract

Lung cancer (LC) is one of the most deadly cancers worldwide, with very low survival rates, mainly due to poor management, which has barely changed in recent years. Nanomedicines, especially gold nanomaterials, with their unique and size-dependent properties offer a potential solution to many challenges in the field. The versatility afforded by the shape, size, charge and surface chemistry of gold nanostructures allows them to be adapted for many applications in the diagnosis, treatment and imaging of LC. In this review, a survey of the most recent advances in the field is presented with an emphasis on the optical properties of gold nanoscale materials and their use in cancer management. Gold nanoparticle toxicology has also been a focus of interest for many years but the studies have also sometimes arrived at contradictory conclusions. To enable extrapolation and facilitate the development of medicines based on gold nanomaterials, it must be assumed that each design will have its own unique characteristics that require evaluation before translation to the clinic. Advances in the understanding and recognition of the molecular signatures of LC have aided the development of personalised medicines. Tailoring the treatment to each case should, ideally increase the survival outcomes as well as reduce medical costs. This review seeks to present the potential of gold nanomaterials in LC management and to provide a unified view, which will be of interest to those in the field as well as researchers considering entering this highly important area of research.

## Introduction

Lung cancer (LC) is one of the four most prevalent cancers worldwide with more than 2.1 million new cases and 1.8 million deaths annually [1]. It is the leading cause of cancer mortality among both men and women, accounting for 25% of all cancer-related deaths. Survival rates vary widely, depending on how far the cancer has spread at the time of diagnosis, which is a key area for improvement [2]. However, the tumour can take years to grow without associated symptoms, making early diagnosis challenging.

Nanotechnology is emerging as one of the most promising ways to improve LC management, mostly through early tumour detection and novel treatment methods capable of selective targeting and delivery of therapeutics. The extraordinary properties of gold nanomaterials promise the potential to deliver remarkable tools for the diagnosis and treatment of LC. For example, their optical properties aid their detection at low concentrations whilst their size and surface modifications can be manipulated in order to achieve significantly improved therapeutic outcomes. This review seeks to provide an insight into how gold nanostructures have become a powerful tool with which to improve current strategies, as well as providing new platforms to overcome the challenges presented by LC.

## LC and gold nanomaterials

### Biology, attributes and types of LC

LC can grow anywhere within the lungs and airways. Smoking leads to the greatest risk of developing LC, being responsible for more than 70% of the cases [2] but it is not the only cause. A recent study highlights how exposure of non-smokers to airborne PM2.5 (particulate matter with an aerodynamic diameter of 2.5 µm or less) correlates with the incidence of LC [3].

Symptoms of LC develop as the condition progresses, but they are rarely detected in the early stages. Signs include persistent coughs and breathlessness that become worse over time, recurrent chest infections, haemoptysis (coughing up blood), tiredness and unexplained weight loss [2].

LC is classified into two main types. Together with the stage of tumour development, this determines which treatments will be recommended. Non-small cell lung cancer (NSCLC) is the most common form, consisting of over 85% of cases, and refers to any type of epithelial cell LC. It can be further divided into three main classes: adenocarcinoma, squamous cell carcinoma and large-cell carcinoma. Small-cell lung cancer (SCLC) represents 10% of all LCs. Compared with NSCLC, it has a shorter doubling time, higher growth fraction and is associated with earlier development of metastases [4].

### Current diagnosis and treatment of LC

**Figure 1.**
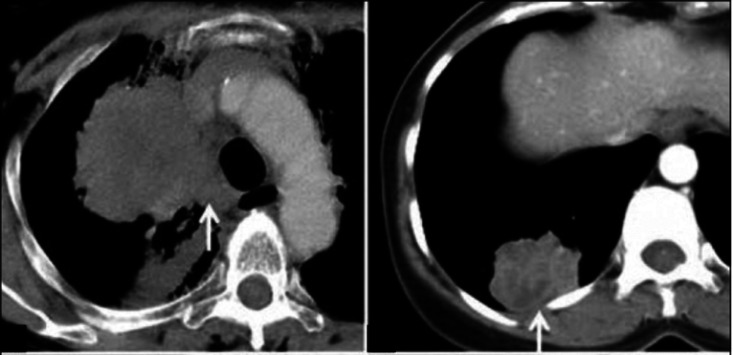
Common radiological appearance of lung cancer. Left panel (arrow): Centrally located mass with mediastinal invasion. Right panel (arrow): Peripherally situated mass abutting the pleura. Reprinted from reference [5] and reproduced with permission.

LC is currently diagnosed using an X-ray scan, where the tumour appears as a white-grey mass. To enable a definite diagnosis and staging of the cancer, further tests are required, such as bronchoscopies, biopsies and ultrasound scans. Figure 1 [5] shows an X-ray of a lung tumour. Current treatment options include surgery, radiotherapy, chemotherapy and immunotherapy, either alone or in combination, and this decision will mainly depend on the stage of the cancer and the overall health of the patient.

Despite recent advances, the diagnosis and treatment of LC is still highly invasive, slow and very expensive. Although surgery is the most successful approach, this still leads to very low long-term survival rates [6]. There is a pressing need to improve early detection as well as reduce the variability in response to current treatments. Nanotechnology offers the possibility of greater precision and more personalised options [7]. The targeting observed for the delivery of intravenously applied nanosized materials to tumours has been attributed to the enhanced permeability and retention (EPR) effect. This phenomenon occurs due to the leaky nature of tumour vasculature, allowing nanoparticles to extravasate into tumour tissue to a higher degree than in healthy tissue [8,9]. It should be noted that the significance and impact of the EPR effect in humans have been the subject of debate particularly in terms of *in vivo* applications. Although many types of nanomedicines exist, gold nanomaterials are starting to demonstrate the benefits of their use, leading to improvements in many techniques, as well as providing a platform for novel therapeutic strategies.

### Gold nanomaterials in LC

**Figure 2.**
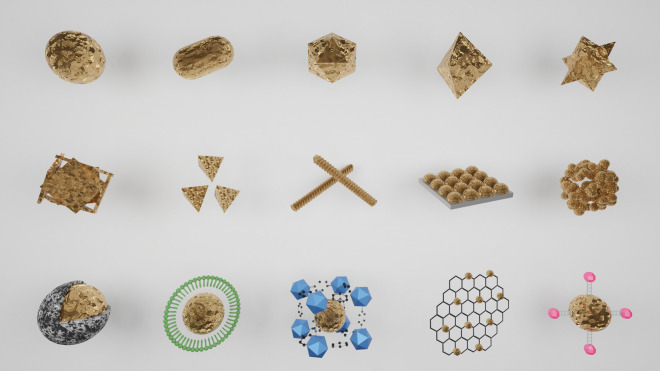
Gold nanomaterials. The first row shows simple gold nanostructures: (from left to right) sphere, rod, polygon, bipyramid and star. The second row depicts nanoscale assemblies based on gold: (from left to right) nanocage, branched nanoparticles, nanohelices, self-assembled layers of gold nanoparticles on surfaces, nanoclusters. The third row shows hybrid nanoscale systems based on gold: (from left to right) silica particles with a gold nanocore, gold nanoparticles inside a polymer vesicle, metalorganic structures encapsulating gold nanoparticles, graphene-conjugated gold nanoparticles and gold nanoparticles with quantum dots. (Original image).

Under most conditions, gold is chemically inert, with a high relative biocompatibility in the human body. Many of its most exciting properties result from reducing its size from the bulk metal to create nanosized materials, which increases their surface area per unit mass, offering a large chemical surface for functional manipulation. In addition to particles resembling spheres, an extensive catalogue of morphologies is available, allowing the function of the nanomaterial to be adapted for a wide variety of applications (Figure 2). For example, rod-shaped structures are preferred in optical applications as they are efficient at absorbing energy from a light source (typically near-infrared light) [10]. For the purposes of this review, the term gold nanomaterial will be used to refer to materials with at least one dimension below 100 nm, irrespective of the specific morphology (nanosphere, nanorod, nanostar, etc.).

**Figure 3.**
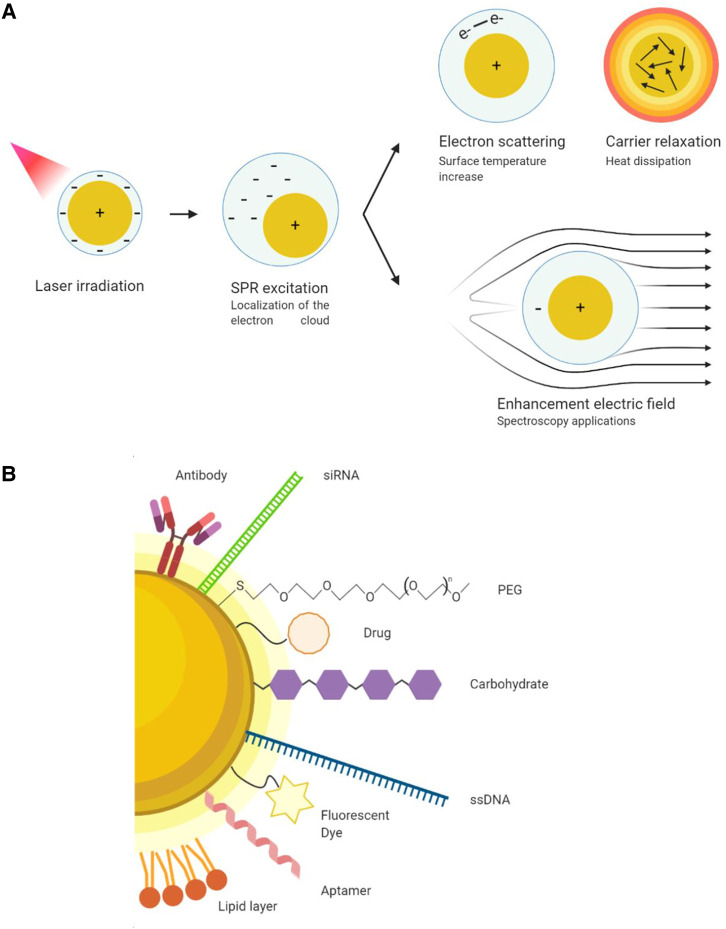
Physicochemical properties of gold nanomaterials. (**A**) Surface plasmon resonance of a plasmonic gold nanomaterial and its applications in therapy, imaging, and diagnostic purposes. (**B**) Gold nanomaterial functionalization strategies and coatings. (Original image).

Due to the size of the nanomaterial, the electrons at the gold surface have the ability to interact with light, resulting in the surface plasmon resonance (SPR) phenomenon. In short, SPR refers to the collective oscillation of conduction electrons in a metal as a result of their excitation by incident light. Influenced mostly by the size and shape of the structure, the SPR is able to modulate the effect of an electromagnetic wave in very localised and focused positions around the material, enabling its utilisation for medical purposes [11] (Figure 3A). With that objective in mind, it is important to tune the SPR absorption wavelength so that it falls within the near-infrared (NIR) region of the electromagnetic spectrum (650–1300 nm). This is known as the ‘biological window’ (or optical/therapeutic window), as light can penetrate more deeply into the tissue due to the absence of absorptions by other biological species in that range [12]. Specific applications related to these optoelectronic characteristics will be discussed in later sections.

The interaction of nanoscale gold materials with biological molecules is mainly determined by surface charge and coating. To prevent aggregation and increase the circulation time in the vasculature, a degree of controllable hydrophobicity is desirable to repel plasma proteins and this is usually achieved through the use of polymer coatings. Polyethylene glycol (PEG) is the most widely used surface coating for gold nanomaterials and a standard strategy to reduce protein corona formation [13] (unwanted adsorption of proteins to the surface of the gold) and to enhance the EPR effect within the tumour [14].

Depending on the desired physicochemical characteristics, different synthetic methods are used to generate the nanostructures, spanning physical, chemical and biological methods. Most approaches generate nanosized gold structures *in situ* with thiol head groups at the metal surface. This enables the amino groups of biomolecules to bind to the terminal carboxylate tail groups extending away from the surface, employing peptide coupling chemistry. Other useful techniques, such as the alkyne-azide cycloaddition reaction (‘click’ chemistry) can also be used, allowing different, orthogonal approaches to be employed for surface functionalisation. This versatility allows the addition of almost any functional group (sugars, antibodies, peptides, proteins, DNA strands, etc.) to the surface of gold nanostructures. Importantly, this permits multiple units to be combined in a controlled manner with the only limitation being available space (Figure 3B). This modular approach to design, together with other favourable properties (compared with other metals), such as the lack of oxide layer and extended intravascular circulation times, make gold nanomaterials ideal candidates for numerous applications in LC management [15–17].

### Toxicology and organ distribution

The same unique properties that make gold nanomaterials so exciting for medical applications can also result in undesirable effects on healthy tissues. It is important to establish whether the nanomaterials are toxic at the concentrations at which they exhibit therapeutic effects, usually in the range of 1–100 particles per cell, as well as how many particles actually enter each cell [18]. Nanosized gold can be redox-active, with the inherent potential to induce the intracellular formation of reactive oxygen species (ROS) that can correlate with toxicity [19,20]. Reported examples of pulmonary cytotoxicity for gold nanomaterials include an increase in cellular invasion [21], epigenetic modifications [22], organelle reorganisation [23] and changes in protein expression [24,25]. Results in this area are sometimes contradictory and dependent on the characteristics of the materials under investigation. However, there is a degree of consensus regarding the role of positive surface charge in the induction of ROS and pro-inflammatory mediators [26]. A study by Elbakary et al. [27] indicated the occurrence of lung remodelling after intratracheal instillation of gold nanoparticles in healthy adult male rats, exhibiting congestion of blood vessels, alveolar collapse, extravasation of red blood cells and thickening in the alveolar wall, suggesting fibrosis. However, the rats were exposed to 40 or 400 µg/kg of the nanomaterial every day for 14 days — doses which might be expected to induce pulmonary inflammation independent of any innate particle toxicity. It is also worth noting that some studies claiming toxicity for gold nanoparticles make use of surface chemistries which are known to be toxic [14], or use unfunctionalized citrate-coated particles [18]. This serves to illustrate that the first interactions between gold nanomaterials and the cells of an organism are through their surface units and so these species will strongly influence the overall toxicity of the material.

The ultimate distribution of gold nanomaterials in the body depends mainly on the administration route employed. Conventional intravenous injection is unlikely to result in delivery to the lung. For example, Koyama et al. [28] found that most gold nanoparticles are located in the liver after tail vein injection, and another intravenous study showed no accumulation of particles in the lung, regardless of particle shape [29]. Similarly, there was little evidence of gold nanoparticles in the lung following oral administration, although there was increased non-lung related toxicity, likely due to the removal of functionalised molecules from the gold surface through the action of gut enzymes [30–32]. Aerosol inhalation or intratracheal inhalation of gold nanoparticles have proved to be the most efficient and safe method for experimental lung delivery applications [33,34]. Although there are differences between these exposure models in terms of the pattern of nanomaterial deposition, safe, relatively non-toxic levels of exposure to the lung can be achieved [34,35]. Thus, most studies of inhaled gold nanoparticles report no harm or signs of inflammation [14] and result in good maintenance of lung integrity and non-deleterious interactions with its biological secretions and fluids [36,37]. Exposure of mice to intratracheal gold nanoparticles of 20 nm diameter resulted in a predominant deposition in the caudal, lower lobes [38]. Approximately 20% of the particles deposited on the large airway epithelium were rapidly cleared by the mucociliary escalator. The remaining 80% were found in the alveoli and relocated from the epithelium to the interstitium of the lung within 24 h, although most of these particles re-enter the airspaces within macrophages and undergo mucociliary clearance. Only a very small proportion (less than 1%) of the material was translocated to other organs, primarily across alveolar type I epithelial cells [39]. Translocation of a very low percentage of metallic, low-solubility nanoparticles across the air-blood-barrier is seen in most inhalation studies [40,41]. The efficiency of the process is to some extent dose dependent [40], but it is also influenced by both the surface charge, which is higher for negatively charged particles [41], and the integrity of the lung (e.g. healthy vs unhealthy) [42]. Surface coatings such as PEG facilitate diffusion through mucus and surfactants in the airways [43]. Although the excretion route of the gold nanomaterials will also depend on their size and shape, there is some agreement that the use of particles between 10 and 100 nm slows down the activation of the mononuclear phagocyte system, while still being large enough to avoid immediate renal filtration [44–46]. It should be noted that in contrast with the study of Elbakary et al. [27], these investigations indicate very little or no toxicity, reflecting the more realistic doses employed.

## Diagnosis using gold nanomaterials

High mortality rates in LC are often a consequence of late diagnosis and there is now a major effort to improve early stage tumour detection, which would lead to better prognosis and survival rates. It is key to identify biomarkers which are overexpressed in the early stages of LC. Gold nanostructures can act as sensors in labelling applications due to their ability to interact with visible light and effectively enhance the signal for diagnostic purposes [47].

### Immunosensors and serum tumour markers

**Figure 4.**
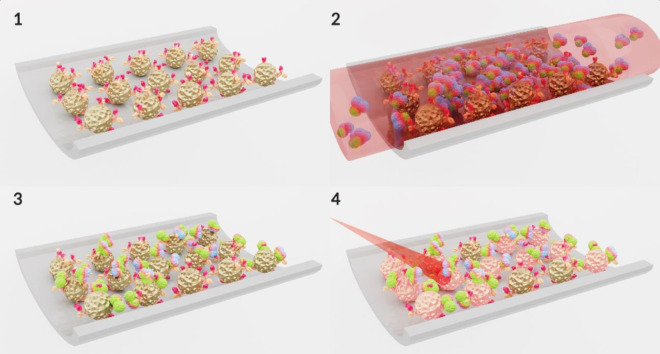
A gold nanoparticle-based detection strategy for biomarkers associated with lung cancer using an immunosensor assay on a microchip. (1) Gold nanoparticles functionalized with a specific lung cancer antibody attached to the microchip channel surface. (2) Gold nanoparticles are exposed to serum from blood of a patient with lung cancer containing the specific biomarker. (3) The sample is washed out and only the specific biomarker remains attached to the antibodies on the GNPs. (4) The attachment of the biomarkers to the GNPs changes their physicochemical character and produces a red shift in their surface plasmon resonance. On irradiation, a characteristic, readable signal is produced that can be used for the diagnosis of lung cancer. (Original image).

Serum tumour markers represent an alternative to invasive methods, not only to improve early diagnosis, but also to monitor the progress of therapy in patients with advanced LC. Immunosensing strategies detect specific antigens overexpressed in tumoural cells and are one of the mostly commonly used diagnostic techniques. However, they often suffer from the need for time-consuming processing and require sophisticated manipulation. Figure 4 shows a representation of a biomarker immunosensing strategy based on gold nanoparticles. Gao et al. [48] reported a straightforward colorimetric assay for the multiplex detection of four LC-associated proteins. This is based on a multilayered approach composed of GNPs functionalised with capture antibodies to generate readable optical signals in a microarray. The immunosensor was able to detect protein concentrations down to 1 ng/ml in serum samples in less than an hour and to combine the detection of four biomarkers. This approach improved the sensitivity of LC diagnosis and staging by up to 88% compared with conventional techniques. Electrochemical biosensors offer an approach that is simple to prepare and use, with fast, label-free detection and inexpensive equipment and materials. These systems usually consist of a sensing substrate with good conductivity over a large surface area and the gold nanomaterials amplify the signal on the electrode surfaces by improving electron transfer. Wang et al. [49] fabricated a conductive hydrogel with electrodeposited GNPs for the sensitive, label-free, detection of neuron-specific enolase (NSE), which is a substance that has been detected in patients with certain tumours. The sensor exhibited a wide linear detection range of 0.001 to 200 ng/ml and a limit of detection of 0.26 pg/ml for NSE, which has a cut-off medical value of 5 ng/ml. Performance comparisons with clinical methods using serum samples reported 93% concordance, indicating substantial clinical promise for such sensors.

### Genosensors

Overexpressed tumour-associated genes represent another set of interesting biomarkers. High- throughput techniques in the field of genomics have recently identified numerous promising microRNAs that play an important role in the development of LC [50]. For example, high miRNA-21 levels are believed to be indicative of lung carcinogenesis status [51]. However, detection of nucleic acids is challenging due to their low abundance and short sequence, making the development of signal amplification approaches critical for their sensing. Specially shaped gold nanomaterials that maximise the surface area are particularly attractive as genosensors. As an example, Su and co-workers [52] developed hierarchical, flower-like gold nanostructures assembled with DNA probes for subsequent hybridisation detection of miRNA-21 resulting in sensitivity as low as 1 fM. Gold nanostructures have also been used to improve the detection of circulating tumour DNA [53], long-noncoding RNAs [54] and other types of microRNAs [55–58] associated with LC.

### Novel sensing approaches

The detection of exhaled volatile organic compounds (VOCs) is considered preferable to biopsies due to the much less invasive nature of the technique. Changes in several VOCs found in exhaled breath have been correlated with the early pathological process of LC, specifically certain aldehydes [59]. Peng and co-workers [60] were the first to report an array of sensors based on gold nanomaterials that could rapidly distinguish between LC patients and healthy individuals based on their breath. More recently, Qiao et al. [61] improved the absorption of gaseous molecules onto a self-assembled layer of GNPs for biomolecular detection of aldehydes in exhaled breath samples, achieving a detection limit of 10 ppb, surpassing by far the sensitivity required for the clinic.

As their role in cancer has emerged in recent years, the detection of exosomes released from LC cells has also been explored using gold nanomaterials. Exosomes are small vesicles (30–150 nm) which are secreted by most types of cells. They have essential roles in tumourigenesis and possess specific membrane proteins, as well as containing proteins, nucleic acids and lipids that regulate malignant biological activity in their mother cells [62]. Methods for sensitive and specific identification of tumour-derived exosomes is being explored for NSCLC, not only for cancer detection but also for metabolic staging and predicting treatment outcomes. In recent work, Fan and co-workers [63] utilised a biosensor to determine populations of NSCLC-derived exosomes using antibody-functionalized GNPs. The strategy was able to distinguish successfully between different LC subtypes and was used to evaluate the therapeutic efficacy by measuring the concentration of exosomes in representative human plasma samples.

## Imaging

High precision imaging is crucial for the early diagnosis and accurate monitoring of LC. The development of new imaging techniques and improvement of current imaging agents is key to targeting cancer in specific locations in the body. Nanosized gold materials offer several advantages over conventional organic dyes and contrast agents due to their low toxicity, spectroscopic properties and negligible quenching [19]. Their ability to accumulate at the site of interest through the EPR effect (see above) is a significant benefit. Their inherent optical properties obviate the need for an applied electromagnetic field, which is important as applied fields can result in the gold nanomaterials actually causing toxicity and tissue damage [64]. The versatility of gold nanomaterials facilitates the combination of multiple imaging modalities to offer complementary information for the accurate monitoring of LC.

### Computed tomography and x-ray scans

**Figure 5.**
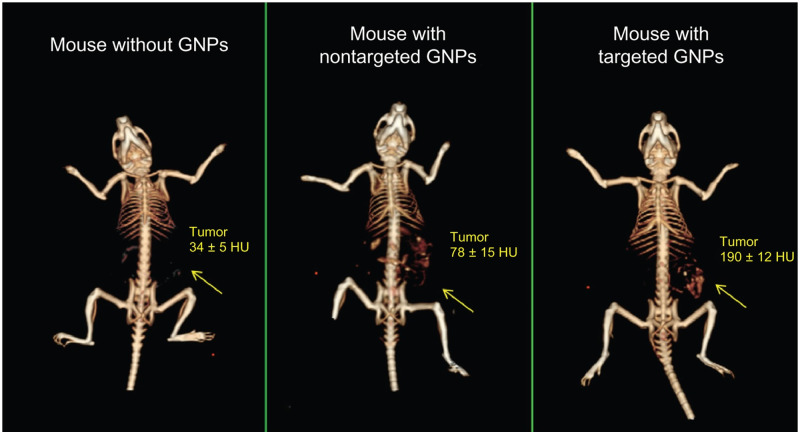
*In vivo* X-ray computed tomography images using a clinical CT scanner. From left to right: (1) Mouse before injection of gold nanoparticles. (2) Mouse 6 h post-injection of nonspecific gold nanoparticles with only passive targeting. (3) Mouse 6 h post-injection of GNPs coated with anti-epidermal growth factor receptor (EGFR) units that specifically target the squamous cell carcinoma tumour. Clear and significant contrast enhancement is visible in (3). Numerical values represent average Hounsfield units (HU) for the whole tumour area. Arrows indicate the tumour site. Reprinted from reference [66] and reproduced with permission.

X-ray and computed tomography (CT) scans are the initial tools used in the diagnosis and staging of LC. A major drawback is the difficulty in distinguishing between tumoural tissue and other lung tissues due to the lack of preferential accumulation of contrast materials in the cancerous cells. The optical properties of gold nanoparticles can enhance the precision of CT images up to 2.5 times due to photoelectric absorption, X-ray attenuation and prolonged circulation time in the lung [65]. The improved contrast also permits the use of a lower radiation dosage to obtain the images, reducing possible side effects. Figure 5 [66] shows an example of this effect. Different doses of gold nanoparticles were used [67] to enhance CT contrast by increasing blood half-life and tumour accumulation in mice-xenografted LC models. An extensive review of the use of GNPs in CT imaging has been published by Ashton and co-workers [68].

### Fluorescence microscopy

The implementation of fluorescence imaging techniques for LC as a medical tool is limited and not widely used. This is mainly due to the toxicity associated with the photobleaching of organic probes and the shallow penetration of tissue associated with this technique. Gold nanoparticles possess extinction coefficients up to 1011 M^−1^ cm^−1^, which is several orders of magnitude higher than those found in organic dyes [18]. The overlap between emission spectra of a given fluorophore and the surface plasmon band of the GNP is known as the fluorescence resonance energy transfer (FRET) phenomenon and can be exploited to enhance the imaging capabilities of this modality [69]. Gold nanoclusters capped with glutathione molecules proved to be non-toxic, exhibiting long intracellular lifetimes (100 ns), and preferentially accumulated in cancerous lung cells when imaged by confocal microscopy [70]. Furthermore, gold nanostructures could be an alternative not only in the clinic but also in pre-clinical laboratory studies. Their use in a pre-clinical setting could overcome some of the limitations of organic dyes, such as poor hydrophilicity and photostability, low quantum yield or insufficient stability in biological systems.

### Magnetic resonance imaging

Magnetic resonance imaging (MRI) is an effective technique used in cancer diagnosis due to its resolution of abnormalities in soft tissues. However, the information it provides is limited in the absence of contrast agents and it suffers from image distortion when imaging the air-blood-barrier due to the presence of large susceptibility changes [71]. Gadolinium chelates bound to nanomaterials improve their performance as contrast agents, through slowing the rotational motion of the chelates and enabling better interaction with endogenous water molecules [11]. For example, [72] dendrimeric Gd-coated gold nanoparticles deliver excellent soft-tissue resolution and increased relaxivity and retention times in HER-2 positive LC in mice. This performance is far superior to that using non-immobilised gadolinium chelates.

### Surface-enhanced Raman scattering

Surface-enhanced Raman scattering (SERS) is a relatively new, ultrasensitive, and non-invasive spectroscopy technique that uses differences in molecular vibration states to distinguish tumoural cells from the surrounding tissue. Enhancements of the SERS signal up to 1015 times have been reported, lowering the detection limit of the technique to the single-molecule level [73]. Gold nanostars (GNS) coated with Nile blue A (a conventional Raman reporter) were investigated [74] in lung adenocarcinoma and alveolar epithelial type-II cells. The SERS spectra could clearly identify important cellular components such as proteins, nucleic acids, lipids and carbohydrates resulting in a characteristic Raman mapping of the tumoural cells. This demonstrated that nanostructures with attached Raman reporter species are able to highlight cellular signatures and provide high spectral specificities on the cellular environment of living cells.

### Treatment of LC using gold nanomaterials

**Figure 6.**
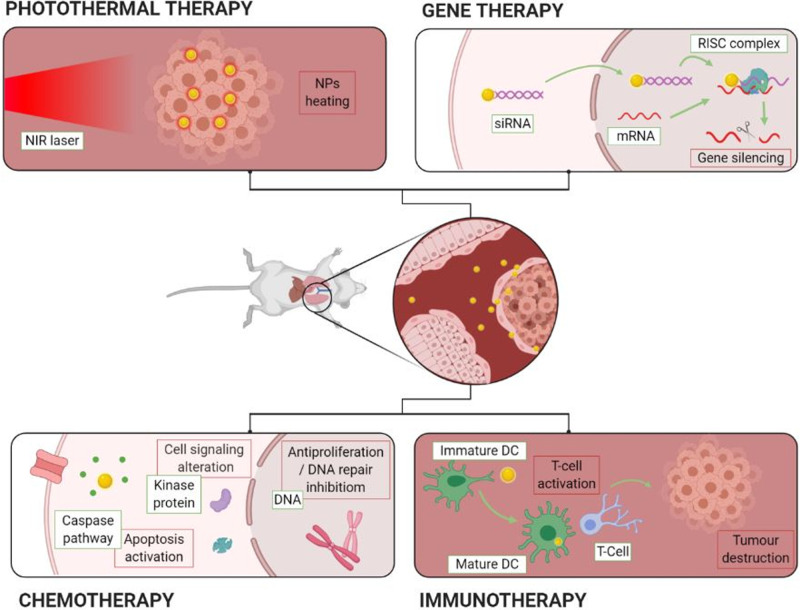
Diagram summarising a range of therapies based on gold nanomaterials including photothermal, gene, chemo- and immuno-therapy. (Original image).

Surgical removal is limited to large, accessible tumours, while chemotherapy suffers from severe side effects and the development of drug resistance. Radiotherapy is an alternative but is highly damaging to healthy tissue along the radiation path. Therefore, novel approaches aim to avoid these drawbacks by using a delivery agent that is cleared from the body once its purpose has been achieved, thereby reducing exposure and limiting toxicity. A graphical overview of the main therapeutic strategies for LC involving gold nanomaterials is shown in Figure 6.

### Improving the delivery of anticancer drugs

Almost all chemotherapeutics are low-density compounds that diffuse rapidly into the tissue and are widely distributed in the body with a short circulatory half-life and undergo rapid clearance [75]. One of the major challenges faced in the field of chemotherapy is the development of resistance by the tumoural cells associated with the low-specificity of the drugs used. Resistance mechanisms include decreased drug uptake, high drug efflux, activation of detoxifying systems and even DNA repair mechanisms [76]. Nanomaterials can be used to target the cancer site, optimise biodistribution of drugs through their ability to translocate across biological barriers, and encapsulate otherwise unstable chemical compounds. Drug-release performance from nanomaterials depends on the strength of the drug attachment or the encapsulation mechanism.

Controlled release also permits a reduction in the quantity of cargo, while still achieving the same clinical response, but with reduced toxicity and improved therapeutic index. For example, activation of mutations in the tyrosine kinase domain of the EGFR gene is the most common genetic abnormality in NSCLC. However, tyrosine kinase inhibitors such as Afatinib suffer from poor tumour accumulation and systemic side effects that limit their clinical use. Cryer and co-workers [77] designed an Afatinib-GNP system to improve drug biocompatibility, successfully achieving a 3.7-fold potency enhancement when administered to LC cells, while maintaining cell viability in a model of a healthy lung epithelium. Another strategy used to limit severe toxic effects and promote a short half-life in the blood is the encapsulation of drugs in pH-dependent nanostructures. A multifunctional gold nanocluster was used by Guo et al. [78] to encapsulate and deliver controllably the antimetabolite drug Methotrexate into a xenograft mouse tumour model. Significant tumour suppression was observed without overt toxicity after 10 days of treatment. Almost all chemotherapy drugs have been successfully conjugated to gold nanostructures, including Methotrexate [79,80], Temozolomide [81], Cisplatin [82], Bortezomib [83], Docetaxel [84], Gemcitabine [85,86] and Doxorubicin [87,88]. The clinical utility of gold nanomaterials is not limited to the delivery of chemotherapeutics but can also be used to increase sensitisation and vulnerability of the tumour before treatment with the drug. For example, CYT-6091, a gold nanomaterial formulation in phase-1 clinical trials, is being used to induce hyper-permeability in the lung and enhance the efficacy of subsequent chemotherapy [89].

### Gene silencing therapy

Antisense DNA and RNA interference have recently emerged as powerful tools to down-regulate gene expression in cancer cells. Small interfering RNAs (siRNAs) can be successfully transfected into cells with controllable strength and duration of the silencing response. These techniques, which work well in the lab, suffer from significant obstacles when applied in the clinic [90]. For example, siRNAs have a short half-life and poor chemical stability, easily dissociate from the vector in a biological environment and, alone, offer weak or no protection against RNases in biofluids. The attachment of nucleic acids to GNPs offers protection against hazardous enzymes and enhances their circulation time in the body as well as permitting tumour targeting when appropriate coatings are used [91]. Many different strategies have used GNPs to knock down transcription factors associated with LC, such as TLR-4/-9 [92], TRAIL [93,94] or IAP-2 [95], amongst others [96,97]. One of the most studied is c-Myc, a key regulator of cell proliferation and apoptosis which, when delivered in association with GNPs, has successfully prolonged survival of lung-tumour bearing mice [98–100]. Recently, Kim et al. [101] used a polymeric multilayer structure with a gold particle core to effectively deliver Myc-siRNA in a bioreductive environment. The formulation was intravenously injected into a murine lung carcinoma xenograph-model and found to significantly suppress tumour growth by 83% compared with the group undergoing no treatment and the group treated with siRNA alone.

### Immunotherapies

The clinical use of biological therapeutics in LC is only occasional and only for patients where the molecular mechanisms/mutations have been established. Other targeted therapies, such as immunotherapy are gaining acceptance for their effective recognition and killing of tumour cells. Immunotherapy involves the activation of the patient's immune system and offers advantages in the inhibition of metastasis and cancer recurrence. Recent research has revealed that gold nanomaterials can be used to activate immune cells as initiators of the immune response. One of the most promising immunotherapy approaches [102] is to promote the maturation of dendritic cells in the lymph node and induce the response of antigen-specific lymphocytes for local LC therapy. In this work, liposome-coated gold nanocages were subcutaneously injected into tumour-bearing mice and a maximal accumulation of the particles was observed in the regional lymph nodes 12 hours post-injection followed by a significant increase in dendritic cell maturation. Although the tumour could not be cleared through immunotherapy alone, its occurrence was significantly delayed, and the strategy is recognised as having potential application in combined therapy.

### Plasmonic therapies

As mentioned briefly above, this section will focus on the use of the plasmonic properties of gold nanomaterials to induce cell damage and kill tumours in LC through the SPR effect [103].

#### Photothermal therapy

**Figure 7.**
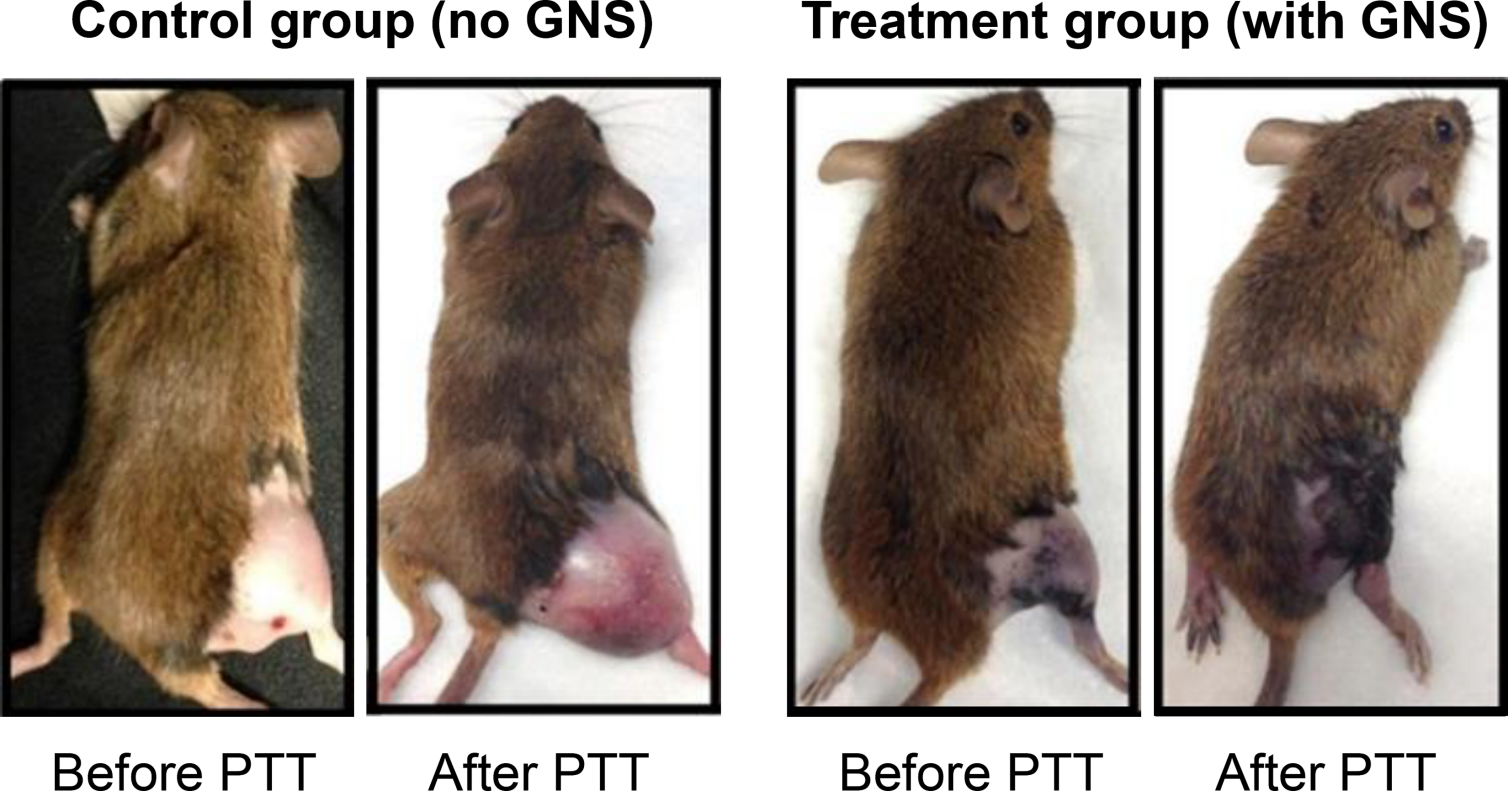
Images of mice before and after photothermal therapy (PTT) with gold nanostars (GNS). The images were taken 3 days after injection with the gold nanomaterial. In the images above, the control group was injected with saline solution. Remission of the tumour in the mice treated with gold nanostars was observed while control mice displayed continued rapid tumour growth. Adapted from reference [12] and reproduced with permission.

Photothermal therapy (PTT) is the most well-known light-driven therapeutic approach and has many promising applications in cancer therapy. It relies on localised induction of heat close to the tumour tissue. Hyperthermia, defined as tissue temperatures above 43 °C, produces irreversible cell damage due to the denaturation of proteins and disruption of cell membranes leading to cellular ablation [104]. After SPR excitation with a specific wavelength of light, a heated electron gas is formed around the gold nanostructure which then cools rapidly (1 ps), exchanging energy with the proximal crystalline network. This leads to energy transfer to the local environment by three processes: electron scattering, electron–phonon coupling and phonon–phonon reaction [105]. Selectivity is achieved by a combination of several factors: focused directional positioning of the incident radiation, efficient targeting of the nanoparticles and the reduced heat tolerance of cancer cells [104]. Figure 7 [12] shows an image of mice before and after photothermal treatment with GNS.

The El-Sayed group has pioneered modern PTT with gold nanomaterials and one of the most relevant studies from this group demonstrated the feasibility of PTT in squamous cell carcinoma in mice [106]. Using gold nanorods (GNR), the preferred shape for PTT due to their enhanced absorption cross-section and two plasmon bands, it was reported that irradiation led to a dramatic decrease in size (*P*<0.0001) of deep malignant lung tumours [106].

Even with mild temperature increases, an acceleration in reoxygenation and perfusion of tumour tissues has been reported to increase chemo- and radiotherapy efficacy, making PTT very attractive as a combination therapy [107]. The promise offered by raised temperatures in conjunction with chemotherapeutics is indicated by a phase II clinical trial, which investigated the effects of two well-known anticancer drugs under mild hyperthermia conditions (NCT00178763). The trial showed an enhanced effect of the therapy after sensitisation with poly(lactic-co-glycolic acid) (PLGA) coated Fe_3_O_4_ nanoparticles [108]. PTT is the main focus of therapeutic approaches based on gold nanomaterials entering clinical trials for LC. Gold-silica nanoshells (AuroShells®) have entered an open-label, single-centre, single-dose efficacy pilot study for the treatment of primary and metastatic tumours of the lung (NCT01679470). In this study, participants were given an intravenous infusion of the PEG-coated nanomedicine followed by laser irradiation of the tumours via fibre optic bronchoscopy. Primary outcomes of the trial reported a decrease in the tumour volume and the finding that participants did not manifest thermal lesions over a 6-month period.

#### Photodynamic therapy

Photodynamic therapy (PDT) uses photoactive molecules (photosensitizers) to induce localised formation of cytotoxic ROS as therapeutic agents. This approach does not use the SPR of gold nanomaterials directly. Instead, their interactions with light enhance and mediate the formation of ROS by amplifying and transferring photonic energy to neighbouring photosensitizer molecules. Some unusual shapes of gold nanostructures have been explored in PDT to enhance the localised electric field around the nanomaterial in the lung. For example, Wang et al. [88] used gold bipyramids to enhance the excitation of a commercially available photosensitizer (AlPcS) 16-fold due to the proximal energy transfer from the surface plasmons of the nanoparticle. Further insights and applications of PDT in LC have been reviewed by Allison et al. [109]. A major drawback of PDT is that it relies on the presence of oxygen to generate ROS but the oxygen concentration at solid tumours is usually limited (hypoxic conditions). Consequently, PDT has been overtaken by PTT in the development of cancer treatment.

## Theranostics

The combination of therapy and diagnostics has led to the new field of theranostics, which seeks to bring together the simultaneous diagnosis, treatment and real-time imaging in ‘all in one’ biocompatible nanomaterials. These multifunctional systems are designed for personalised and specific LC management by merging chemical and physical properties into a single material. For example, Liu et al. [87], developed multioperative GNR encapsulating Doxorubicin and gadolinium surface units for combined chemotherapy/PTT/MRI applied to EGFR-positive tumours. The assembly displayed enhanced contrast images and promoted the destruction of tumoural cells after laser activation *in vivo*. No signs of morphological damage to healthy tissues were reported, indicating reduced side effects of the Doxorubicin drug. Another less common and highly dangerous LC mutation was efficiently treated through the application of a theranostic approach. Anaplastic lymphoma kinase (ALK) tumours represent around 8% of NSCLC. The routinely used ALK-targeted drugs have been shown to induce mutations that lead to drug resistance after 8 months of chemotherapy. Li et al. [110] devised a dual-target siRNA/PTT/chemotherapeutic assembly based on a gold nanoshell system that significantly improved drug delivery to the tumour *in vivo*. Upon laser irradiation, significant ALK-gene inhibition occurred only at the tumour site. The plasmonic properties of gold nanomaterials have been exploited by combining PDT/PTT with photoacoustic/NIR fluorescence imaging in a single GNS system [111]. The functionalised nanostars were able to target the cancer specifically in lung xenograph tumour-bearing mice. Tumour growth was significantly suppressed (by 93% compared with controls) with negligible damage to secondary organs and no variation in the body weight of the animals. In addition to these effects, the fluorescence intensity of the images was enhanced more than 3-fold. These examples and many more currently being developed hold significant clinical potential for nanogold applications to drive LC treatment with the ultimate aim of personalising detection and treatment for each patient.

## Conclusions

LC has one of the highest mortality rates of all cancers with conventional methods requiring invasive procedures and non-invasive alternatives, such as chemotherapy and radiotherapy, which suffer from substantial side effects. There is, therefore, an urgent need for new diagnostic, imaging and treatment tools. Gold nanostructures have emerged as a powerful vehicle that can be used to overcome solubility and stability issues of drugs, reduce mis-targeting of the tumour and overcome biological barriers that previously made treatment difficult. The shape, size and surface chemistry of gold nanomaterials are the key factors that allow these materials to be adapted to enhance the aforementioned applications in medicine. The manipulation of their structure and their surface functionality permits optimised cellular targeting, internalisation and many favourable biomolecular interactions. Nevertheless, a clear understanding of the distribution of the particles inside the body, their interplay with healthy tissue and, above all, their clearance mechanisms, will determine whether they are safe for use in humans. The literature on the toxicology of gold nanomaterials is substantial and covers many diverse aspects. It is clear that every single nanogold design has its own unique properties (e.g. size, shape, charge, surface chemistry, etc.) and so there is a pressing need to unify concepts to enable extrapolation to the clinic and facilitate the development of diagnosis and therapy based on these promising materials. The latest studies in the field identify inhalation as the best way to deliver gold nanomaterials to the lung, indicating high deposition efficiency, good distribution, maintenance of the tissue integrity and no signs of induced inflammation. In recent years, high precision techniques in gene sequencing and -omics technologies have revolutionized our knowledge of LC, helping to understand its molecular signatures. This increase in information on the mechanisms involved in LC has led to better identification of biomarkers that can be translated into more precise and faster diagnostic techniques. Biosensor devices based on gold nanomaterials are promising for research purposes and ultimately for use as non-invasive diagnostic tools for early detection, which is a key factor in the battle against LC. In terms of treatment, gold nanostructures with a high surface area offer greater targeted drug payload, permitting a reduction in the drug intake, which in turn translates into less systemic side effects. As discussed above, gold nanomaterials are also promising therapeutics in their own right, for example, when stimulated by an appropriate light source. Photothermal therapies have demonstrated their effectiveness and safety and are leading the way in the translation of nanogold-based treatment to the clinic. Another promising area is the coupling of photo-responsive agents to the surface of the gold nanostructures to enhance the performance of almost any light-based imaging technique. This is particularly useful in the field of theranostics, where the diagnosis, treatment and imaging of LC could be achieved simultaneously using a single construct.

While this is very encouraging, any consideration of this field must be realistic and accept that most studies are still in their infancy. The majority of the approaches presented in this review, although exciting, are in the initial phases of development and have not yet found their way into the clinic. Unlike other FDA-approved gold nanomedicines, such as Aurolase® PTT therapy for prostate cancer [112], the sensitivity of the lung makes the delivery of therapeutics particularly challenging. As highlighted above, there are many issues that have to be overcome before clinical translation can be achieved. Nevertheless, these early innovative studies indicate a broad range of applications for gold nanomaterials as anticancer weapons in the future, including for LC, either alone or in combination with other techniques. Ultimately, their success will depend on continued investment and research into their development. These aims will benefit from greater consensus and cohesion within the field, drawing together advances in big data, nanomedicine, optics, analytics and supramolecular chemistry.

## Summary

- Gold nanomaterials offer a wide variety of attributes that allow them to be adapted to permit or enhance diagnosis, staging and treatment of LC
- Greater integration of the concepts would enhance translation to the clinic and facilitate the development of therapies based on gold nanomaterials.
- Emerging fields such as theranostics hold great promise for the development of new gold-based nanomedicines.
- Although the approaches presented here are still in their infancy, they clearly point towards the use of gold nanomaterials as anticancer weapons of the future.

## Open Access

Open access for this article was enabled by the participation of Imperial College London in an all-inclusive *Read & Publish* pilot with Portland Press and the Biochemical Society under a transformative agreement with JISC.

## Abbreviations

ALK: anaplastic lymphoma kinase
CT: computed tomography
EPR: enhanced permeability and retention
GNR: gold nanorods
GNS: gold nanostars
LC: lung cancer
MRI: magnetic resonance imaging
NIR: near-infrared
NSCLC: non-small cell lung cancer
NSE: neuron-specific enolase
PDT: photodynamic therapy
PEG: polyethylene glycol
PTT: photothermal therapy
ROS: reactive oxygen species
SCLC: small-cell lung cancer
SERS: surface-enhanced Raman scattering
SPR: surface plasmon resonance
VOCs: volatile organic compounds
